# Gestational diabetes promotes germ cell cCyst breakdown and primordial follicle formation in newborn mice via the AKT signaling pathway

**DOI:** 10.1371/journal.pone.0215007

**Published:** 2019-04-11

**Authors:** Junjun Xu, Jiaojiao Huang, Qingjie Pan, Miao Du, Zhen Li, Huansheng Dong

**Affiliations:** Department of Animal Science and Technology, Qingdao Agricultural University, Qingdao, China; China Agricultural University, CHINA

## Abstract

Type 1 diabetes (T1D) is a common disease in which pancreatic β cells are impaired due to auto-immunity, pregnancy in women with it is associated with increased risk of neonatal morbidity, mortality. However, the effects of gestational diabetes on the reproduction of newborn offspring are still poorly understood. Here, we determined the cyst breakdown and primordial follicle formation in neonatal offspring born by streptozotocin (STZ)-induced diabetic or non-diabetic female mice, and found that the germ cell cyst breakdown was promoted in neonatal offspring of STZ -induced diabetic mice at postnatal Day 1, which sequentially accelerated the primordial follicle formation. Further investigation revealed that, the expression level of PI3K and p-AKT were significantly increased in ovaries of offspring born by T1D mice. These results indicated that STZ -induced gestational diabetes promotes germ cell cyst breakdown and primordial follicle formation by regulating the PI3K/AKT signaling pathway in the newborn offspring. In addition, this effect can be rescued by an insulin supplement. Taken together, our results uncover the intergenerational effects of gestational diabetes on neonatal offspring folliculogenesis, and provide an experimental model for treating gestational diabetes and its complications in neonatal offspring.

## Introduction

Type 1 diabetes (T1D) is a common disease in which pancreatic β cells are impaired due to auto-immunity [1], accounts for 5–10% of all diabetes, and typically occurs in children and adolescents especially in developed countries [2]. T1D has recently been on the rise globally. Nearly half of pregnant women are either overweight or obese at conception [3]. These adverse conditions significantly increase the risk of gestational diabetes in pregnant women [4]. The occurrence and development of pregnancy complications are closely related to the blood glucose levels in women with T1D [5]. Pregnant women with T1D display a higher incidence of reproductive problems, such as infertility, miscarriage, and their offspring are more likely to develop congenital malformations and show a higher rate of fetal death than those of nondiabetic mothers [6–8].

In mammals, germ cells cyst breakdown, primordial follicle formation, and primordial follicle pool establishment are vital for the function of oocytes [9]. Throughout their migration, primordial germ cells (PGCs) arrive at the gonad around embryonic day 10 (E10) in mice and mitotically divide with incomplete cytokinesis to form cells called nests or germ cell cyst [10]. For female fetuses, PGCs undergo meiosis at approximately E13 in mice [11]. Oocytes are arrested in the diplotene stage of meiotic prophase I at birth [12] and exists as cyst [13]. Until birth, the oocyte cyst breaks down and separates into individual oocytes which are surrounded by a single layer of granulosa cells to form primordial follicles [14].

Previous studies showed that maternal diabetes adversely affects oocyte quality and pre- and post-implantation embryonic development [6, 15, 16]. Maternal diabetes makes preovulatory oocytes to experience a delayed germinal vesicle (GV) breakdown and changes cellular metabolisms such as mitochondrial malfunction and abnormal glucose metabolism [17–19]. Oocytes in diabetic mice display a higher frequency of spindle defects and chromosome misalignment in meiosis, resulting in a significantly increased incidence of aneuploidy in ovulated oocytes [20]. Evidence suggests that maternal diabetes disrupts the endoplasmic reticulum (ER) distribution pattern, leads to abnormal dynamic changes of ER during oocyte maturation and early embryo development in mouse [21]. Furthermore, maternal diabetes was shown to have adverse effects on DNA methylation of maternally imprinted gene Peg3 in mouse oocytes in a time-dependent manner [22]. To restore the abnormal development caused by T1D, at present, blood glucose levels of pregnant women are suggested to control with the help of dieting and insulin treatment. [23, 24].

Maternal diabetes has adverse effects not only on oocyte quality but also on embryo development. However, the effects of pregnant women with T1D on the development of follicles in newborn offspring remains poorly understood. The objective of this study was to examine how T1D during pregnancy affects the follicles quality and development in newborn offspring. In this study, the effects of high blood glucose during pregnancy on early follicular development of newborn offspring were analyzed by establishing the mouse model of gestational diabetes mellitus (GDM), to providing theoretical basis for treating GDM.

## Materials and methods

### Animals

All animal procedures described in the present study were reviewed and approved by the Ethical Committee of Qingdao Agricultural University. ICR mice (Vital River, Beijing, China) used for all experiment were maintained on a 12-h light, 12-h dark cycle (lights off at 20:00 h) with food and water available ad libitum. Animals were cared for in accordance with all national and institutional guidelines.

### Induction of diabetes and treatment

**Fig 1:**
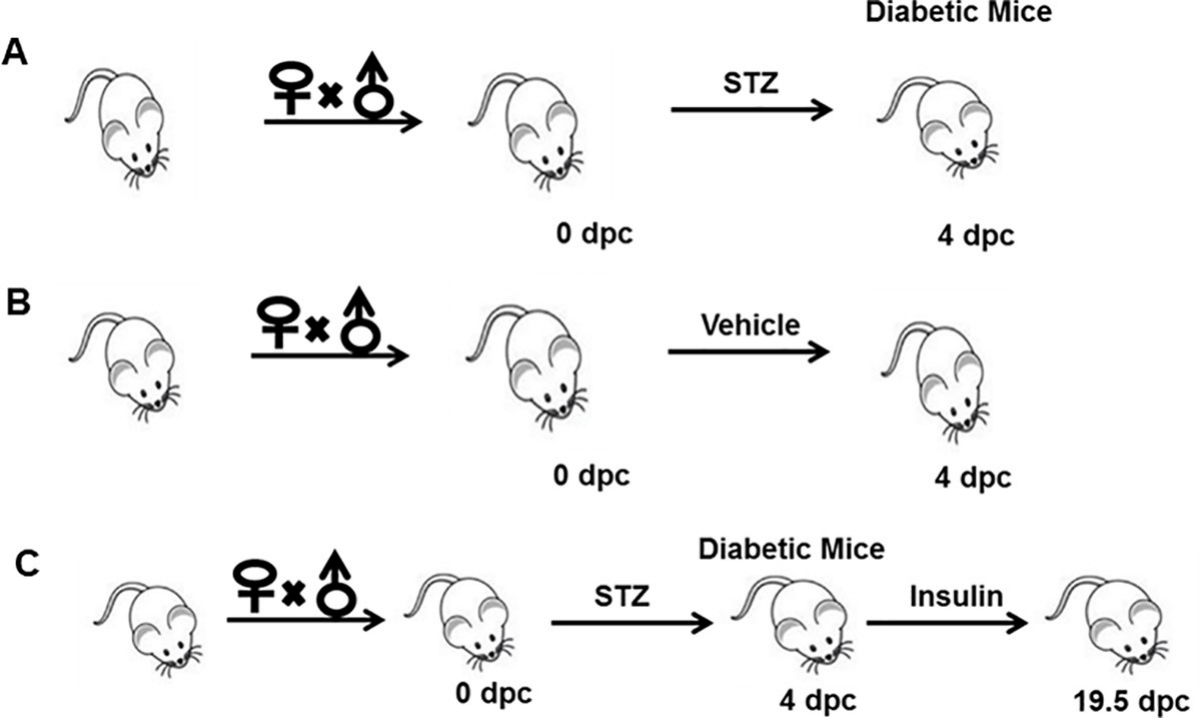
Mating, generation of diabetic mice and insulin treatment. **(A)** Female ICR mice were mated with wild type male mice to achieve pregnancy. Mice on day 1 of pregnancy received a single injection of STZ at a dose of 200 mg/kg to induce diabetes. Four days later, blood glucose levels were measured via a commercial glucometer. Mice with glucose levels that were greater than 260 mg/dl were considered to be diabetic. **(B)** Control mice received an equal volume of the sodium citrate vehicle buffer. **(C)** After we determined that the mice were diabetic, 2 IU insulin (Sanofi-Aventis, Germany) was injected into diabetic mice until fetal birth. At least 6–8 mice were included in each group. dpc, means day poist coitum.

Mating was timed overnight and the appearance of vaginal plug in female ICR mice (age 6–7 weeks) was considered as pregnancy the next morning. To generate the diabetic mouse model, pregnant female ICR mice received a single injection of streptozotocin (STZ) at a dose of 200 mg/kg. Four days after injection, a tail-blood sample was measured for glucose concentrations via a Hemocue B glucose analyzer (Stockholm, Sweden) once a week. If glucose levels were greater than 260 mg/dl, the animal was selected for use as a diabetic model (Fig 1A). For the insulin treatment group, 2 IU insulin (Sanofi-Aventis, Germany) was injected into diabetic mice twice at 12:00 and 24:00 from day 4 after STZ injection until fetal birth. Following insulin injection, glucose level was monitored from day 5 once every week to ensure that blood glucose returned to normal level (Fig 1C). Female ICR mice of similar age injected with the sodium citrate vehicle buffer were selected as control animals (Fig 1B).

### Histology and morphological evaluation ovaries

Ovaries tissues from the newborn offspring in both treated and control groups at PND 1 were fixed in 4% paraformaldehyde (PFA, Beyotime) overnight, transferred to gradients of alcohol and embedded in paraffin. A series of 5 μm sections were stained with hematoxylin and eosin (Sigma) for histology and morphological evaluation ovaries following the standard procedure.

### Immunofluorescence

Ovaries were cleaned and washed with PBS 3 times, then fixed in 4% paraformaldehyde at 4°C overnight. The ovaries were treated with histological procedures, paraffin embedding and were sectioned into 5 μm sections. These sections were held at 60°C for 30 min, and placed in xylene and rehydrated. Then, sections were transferred into sodium citrate buffer at 96°C for 10 min. Following 45 min of blocking with BDT (TBS with 10% goat serum and 3% BSA), sections were incubated with primary antibodies at 4°C overnight. Then sections were incubated with Cy3-labeled or FITC-labeled secondary antibodies (dilution 1: 150, Beyotime, A0516, China) for half an hour at 37°C. Counterstaining was treated with Hoechst33342 (Beyotime) for 5 min. Vectashield (Vector, H-1000) was used to seal the coverslips. Then the sections were analyzed under Olympus BX51 fluorescence microscope (Olympus, Japan).

### Follicles counting

To determine the number of cysts and primordial follicles, every fifth section was counted for analysis. Intact oocytes were detected if there are no surrounding granulosa cells or if they were stained positive for MVH. Primordial follicles were classified as an oocyte partially surrounded by squamous and cuboidal granulosa cells.

### Quantitative real-time PCR (RT-qPCR)

Ovaries were derived from the newborn offspring in both treated and control groups at PND 1. Total RNA was extracted using RNAprep pure Micro Kit (Aidlab, RN07) according to the manufacturer’s instructions. After isolation, extracted RNA was exposed with DNase, and then reverse transcription was conducted using the FastQuant RT Kit (With gDNase) (TIAGEN, Beijing, China). The synthesized cDNA was used for quantitative real-time PCR. The housekeeping gene, β-action, was used as an internal control (primer sequences are shown in Table 1).

**Table 1:** The related primers for quantitative-PCR.

Gene	Primers	Length (bp)	Gene no.
*Nobox*	F:5′-CTATCCTGACAGTGACAAACGCC-3′	251	NM_130869.3
	R:5′- CACCCTCTCAGCACCCTCATTAT -3′		
*Figla*	F:5′- ACAGAGCAGGAAGCCCAGTA-3′	225	NM_012013.1
	R:5′- TGGGTAGCATTTCCCAAGAG-3′		
*Sohlh2*	F:5′- TCTCAGCCACATCACAGAGG-3′	199	NM_028937.3
	R:5′- GGGGACGCGAGTCTTATACA-3′		
*Lhx8*	F:5′- CAGTTCGCTCAGGACAACAA-3′	105	NM_000069.5
	R:5′- CCTGCAGTTCTGAAACCACA-3′		
*Vasa*	F: 5′-AGCTGGGACATTCAATTCGAC-3′	220	NM_001166534.1
	R:5′-GTTTGGCTGCGTTCCTTTGAT-3′		
*Kitl*	F:5'-AATCCTCTCGTCAAAACTGAAGG-3'	163	NM_000899.4
	R: 5'-CCATCTCGCTTATCCAACACTGA-3'		
*InsR*	F 5’- CAAACAGATGCCACTAATCC- 3’	185	ENSMUST00000207100.1
	R 5’–CTTTGAGACAATAATCCAGCTC- 3’		
*PI3K*	F: 5′- ACACCACGGTTTGGACTATGG-3′	140	ENSMUST00000190171.1
	R: 5′- GGCTACAGTAGTGGGCTTGG-3′		
*β-action*	F:5′- TCGTGGGCCGCTCTAGGCAC-3′	255	NM_007393.3
	R:5′- TGGCCTTAGGGTTCAGGGGG-3′		

The PCR was conducted using TaKaRa SYBR Premix Ex Taq (TaKaRa, Tokyo, Japan). Quantitative PCR was carried out with LC 480 SYBR Green I Master in a LightCycler 480 system (Roche). Primer validation tests were conducted for each designed primer to verify that the amplification efficiencies were similar for each cycle. The program used for the PCR included an initial temperature of 95°C for 30 s, followed by 40 cycles at 95°C for 5 s and 60°C for 34 s. Real-time fluorescence data were collected during the extension time. The relative quantification method based on the comparative values for the threshold cycles (Ct) was used to identify the abundance of the message. The transcript abundance of each gene was calculated relative to that of the internal control gene *β*-actin. ΔCt was calculated by subtracting the Ct values of each gene from the Ct values for *β*-actin. The control group Ct values served as calibrators and were subsequently used to obtain ΔΔCt values. Fold differences in transcript abundance were obtained using the equation 2^-ΔΔCt^. At least three biological and three experimental replicates were used for each assay. The quantitative real-time PCR results were compared through one-way ANOVA using the Statistical Analysis System software SAS 6.12 (SAS Institute, Cary, NC). Differences with p<0.05 were considered significantly different.All experiments were repeated 3 times and values shown are the mean ± SEM.

### Western blot analysis

Proteins were extracted from ovaries tissues with RIPA (Beyotime, P0013C) lysis buffer for 30 min on ice with frequent vortexing. SDS-PAGE sample loading buffer was added into the lysate which was subsequently boiled for 5 min. Then lysates were collected by centrifugation at 14,000 rpm for 5 min. Total proteins were separated by SDS-PAGE with a 5% stacking gel and a 10% separating gel for 50 min at 100 V and 2.5 h at 120 V, respectively, and then electrophoretically transferred onto PVDF membrane. After blocked at room temperature in TBST buffer containing 10% BSA (Sigma), the membranes were hybridized with primary antibodies overnight at 4°C. Finally, the membranes were washed with TBST for three times, then incubated for 2 h at room temperature with secondary antibody horseradish peroxidase (HRP)-conjugated goat anti-rabbit IgG (Beyotime, A0208) at a dilution of 1:1000 in TBST. Membranes were washed three times for 5 min each in TBST. Signals were analyzed by the enhanced chemiluminescence (ECL) detection system. The beta Actin antibodies were detected as a control. Primary antibodies including rabbit anti-AKT polyclonal antibody (Abcam, ab86926), rabbit anti-AKT (phospho S473) polyclonal antibody (Abcam, ab66138), rabbit anti-beta Actin polyclonal antibody (Abcam, ab8227) were used in this study.

### Statistical analysis

For each result, independent experiments were repeated at least three times. Data are presented as means ± SEM. The differences between treated groups and controls were analyzed by ANOVA, and differences were calculated by Tukey’s test. *P*<0.05 was considered statistically significant.

## Results

### Effects of maternal diabetes on the development of offspring

**Fig 2:**
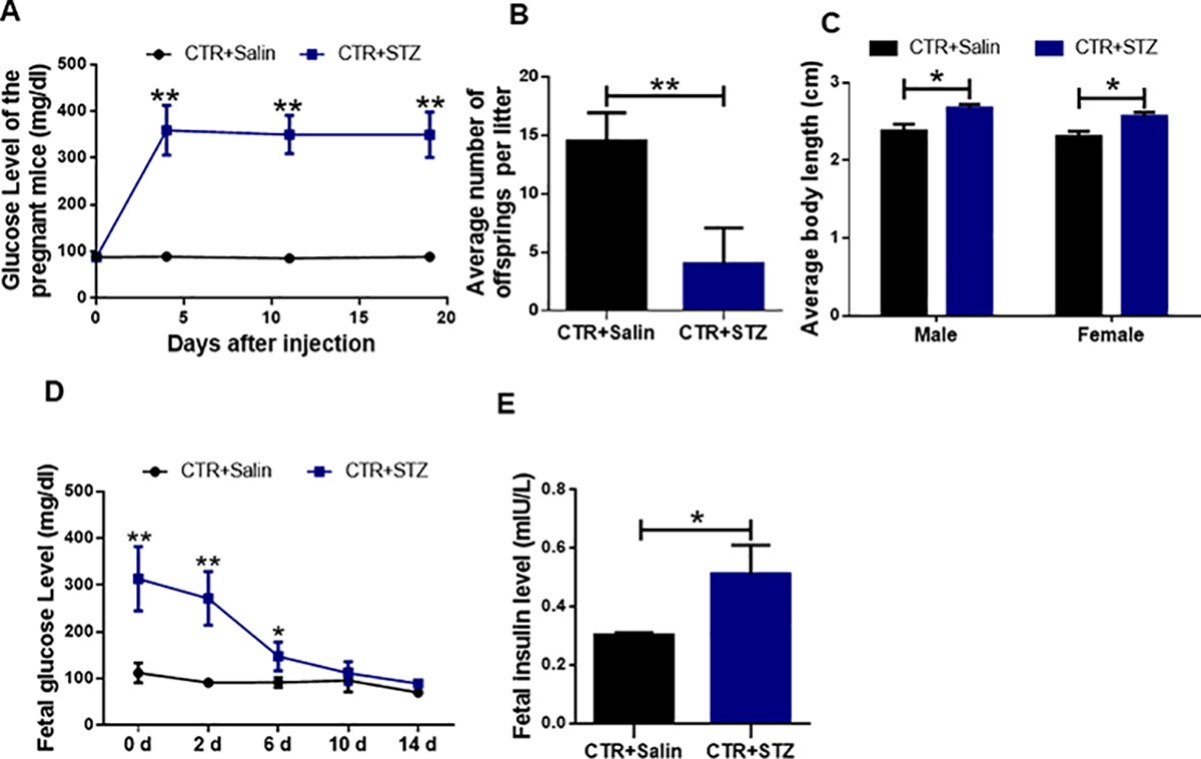
Effects of gestational diabetes on the development of newborn mice. **(A)** The glucose concentrations in STZ-treated and control mice in 3 weeks. The average glucose concentrations in STZ-treated and control mice at different time points were counted. Date are presented as mean ± SEM. **(B)** The average number of newborn mice in STZ-treated and control groups. **(C)** The average length of newborn mice in STZ-treated and control groups. **(D)** The glucose level of newborn mice in STZ-treated and control groups in two weeks. **(E)** The serum insulin level of newborn mice in STZ-treated and control groups. At least 8–10 mice were included in each group; Date are presented as mean ± SEM. *shows a significant difference between STZ-treated and control at P < 0.05; ** shows a significant difference at P < 0.01.

Pregnant female ICR mice received a single injection of STZ at a dose of 200 mg/kg. Female mice of similar age injected with the sodium citrate vehicle buffer were selected as control animals. Four days after injection, we measured glucose concentrations once a week. The glucose levels were greater than 260 mg/dl in STZ group, as shown in Fig 2A, and then the animals were selected for use as a maternal diabetic model. Maternal diabetic mice developed increased glucose concentrations in 3 weeks. And we found that the average glucose concentration was significantly higher in the STZ-treated group compared to the control (*P* < 0.01), suggesting a successful establishment of a diabetic pregnant mouse model.

Our previous studies have demonstrated that maternal diabetes adversely affects preimplantation embryo development and pregnancy outcomes, but whether gestational diabetes affects fetal development is poorly understood, so we investigated the effects on the development of newborn offspring. We collected newborn offspring at PND 1 and results showed that the average number of offsprings was 14.8±2.6 in the control group. However, it was 4.2±3.2 in the STZ-treated group, and there were significant differences between the control and STZ-treated groups (P < 0.01) (Fig 2B). This result recapitulated what has been observed in pregnant women with T1D who suffered miscarriage and marked increase of the newborn death. The mice were anesthetized and then the body length was measured from the head to the beginning of the tail [25]. As shown in Fig 2C, our statistics analysis found that the average length was longer in the STZ-treated group compared to the control (*P* < 0.05). Then we measured offspring serum glucose and insulin levels. We found that fetal glucose and insulin levels were significantly higher in the STZ-treated group compared to controls (P<0.05) (Fig 2D and 2E), but returned to normal levels in about two weeks. Our studies demonstrated that gestational diabetes adversely affects the newborn offspring development.

### STZ induced gestational diabetes promotes germ cell cyst breakdown and primordial follicle formation

**Fig 3:**
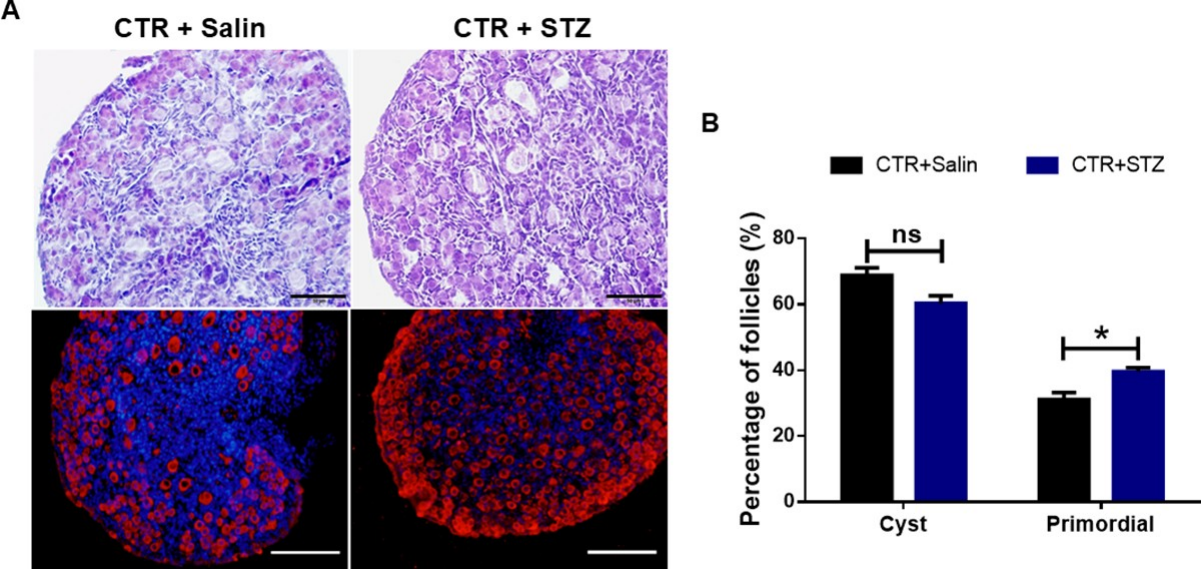
Analysis of follicle development in newborn mice of STZ-induced gestational diabetes. **(A)** It showed the histological appearance of the newborn mouse ovarian tissue (Scale bars: 50 μm) and ovary whole-mount substratesimmunofluorescence (Scale bars: 100 μm) with MVH antibody in control and STZ-treated groups at PND 1. Oocytes are stained with MVH (red) and Hoechst33342 (blue) to label the nucleus. **(B)** It showed the percentage of oocytes in cyst and primordial follicle in control and STZ-treated groups at PND 1. At least 5 mice were analyzed in each group; Date are presented as mean ± SEM. *shows a significant difference between STZ-treated and control at P < 0.05; ** shows a significant difference at P < 0.01.

Previous studies have shown that STZ-induced diabetes adversely affected the preovulatory oocyte maturation and the development of the perinatal period in mouse, but whether STZ-induced gestational diabetes affects follicle development in newborn mice is poorly understood. In order to examine the effects of STZ-induced T1D on the breakdown of germ cell cyst and primordial follicle formation, we calculated the number of oocytes that were still in the cysts and the number of primordial follicles in STZ-treated and control newborn mice ovaries at PND 1 using HE staining and ovaryimmunofluorescence. No obvious change in total oocytes was observed between STZ-treated and control groups. However, there was a significant difference in oocytes of the cyst or primordial follicles between the STZ-treated and control groups (Fig 3A). After calculation, the percentages of unassembled follicles and primordial follicles were 68.82 ± 2.36% and 31.18 ± 2.10%, respectively, in control group; while they were 60.39 ± 2.33% and 39.61 ± 1.25%, respectively, in STZ-treated group (Fig 3B). These results indicate that STZ-induced gestational diabetes accelerates germ cell cyst breakdown and primordial follicle formation in newborn mice significantly (Fig 3B).

### Gestational diabetes increase the mRNA Level of primordial follicle assembly related genes

**Fig 4:**
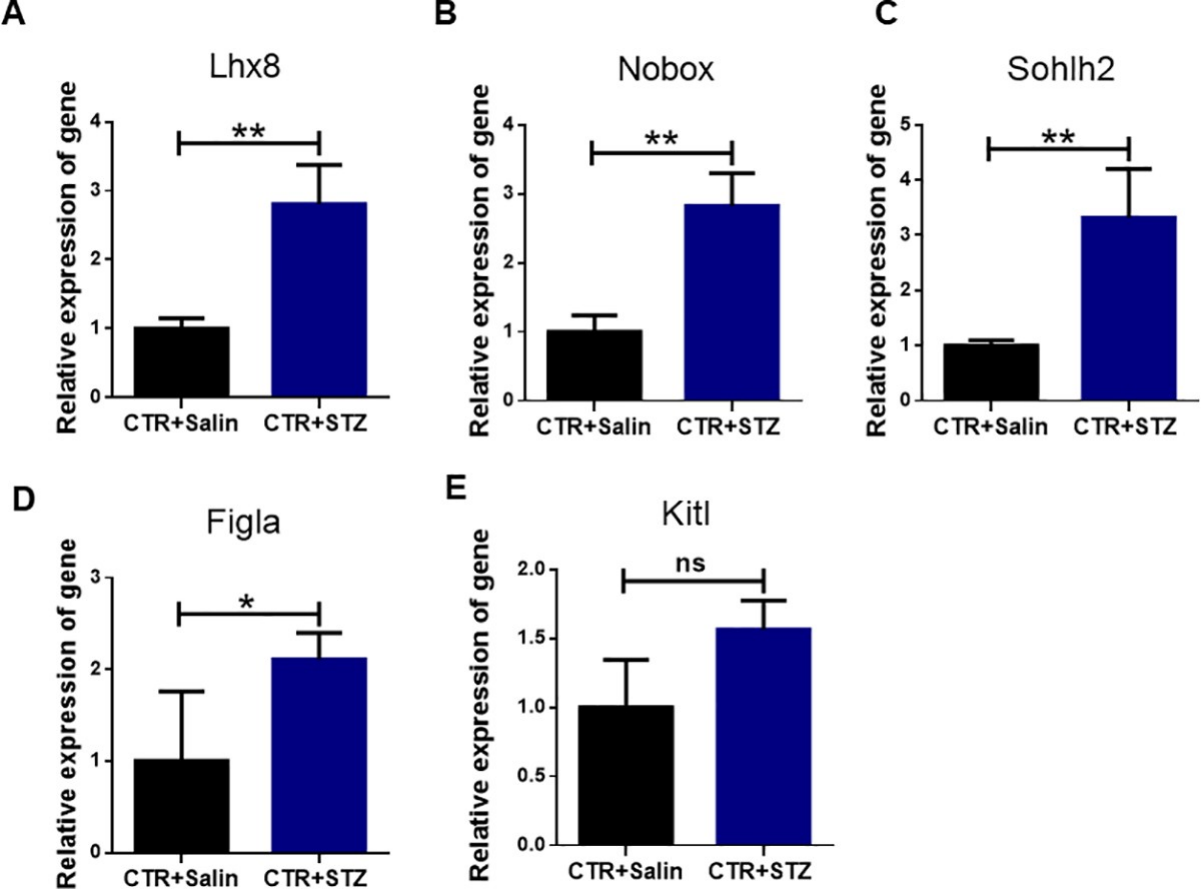
Real-time PCR analysis mRNA expression levels of oocyte-specific genes in control and STZ-treated group in newborn mouse ovaries. **(A-E)** Relative expression levels of oocyte-specific genes including Lhx8, Nobox, Sohlh2, Figla and Kitl in newborn mouse ovaries derived from the control and STZ-treated groups. The expression levels were normalized against β-actin. Noteworthy, STZ treatments in the pregnant mice significantly increase the expression level of Lhx8, Nobox, Sohlh2 (P<0.01) and Figla (P<0.05) in the newborn mouse ovaries. Samples from 5 individual mice were analyzed.

To further confirm the effects in primordial follicle formation caused by gestational diabetes, the mRNA of oocyte specific genes such as LIM homeobox 8 (Lhx8), newborn ovary homeobox (Nobox), spermatogenesis and oogenesis helix-loop-helix (Sohlh2), and factor in the germline alpha (Figlα) were examined in ovaries of neonatal offspring at PND 1. As shown in Fig 4, the mRNA expression levels of these genes were significantly higher in the STZ-treated group compared to the control (Fig 4A–4D). Interestingly, the expressions of Kitl, an important granulosa cell-derived growth factor in ovarian folliculogenesis[26], showed no significant difference between control and STZ-treated groups (P>0.05) (Fig 4E). These results demonstrate that the assembly process of mouse primordial follicles are promoted in the ovaries of neonatal offspring at PND 1.

### PI3K-AKT signal pathway modulates the newborn offspring follicle development

**Fig 5:**
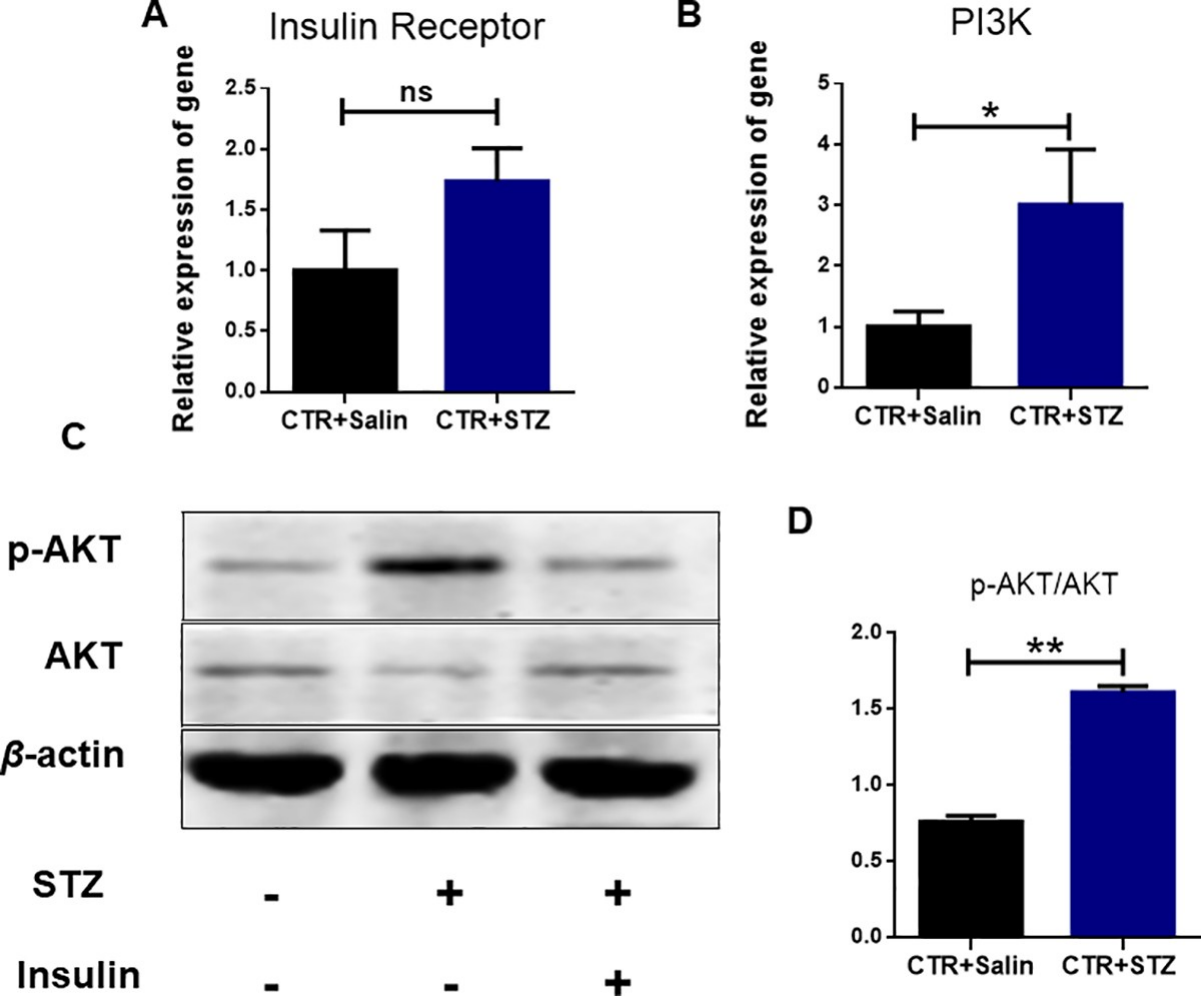
STZ-induced gestational diabetes upregulates the PI3K signaling pathway in newborn mouse ovaries. **(A, B)** Real-time PCR analysis of the mRNA expression levels of insulin receptor and PI3K gene in control and STZ-treated groups in newborn mouse ovaries.(**C, D)** The expression of AKT and p-AKT proteins were analyzed by western blot. Expression of β-actin was used as a loading control. Data was the ratio of p-AKT and AKT. Samples from 5 individual mice were analyzed. Date are presented as mean ± SEM. *shows a significant difference between STZ-treated and control at P < 0.05; ** shows a significant difference at P < 0.01.

Previously, it was reported that folliculogenesis was stage-specifically regulated by insulin via the Akt signaling pathway in vitro[27]. To investigate the developmental activation process of mouse primordial follicles, the expression of insulin receptor, PI3K and phosphorylation of AKT was examined in the ovaries of neonatal offspring at PND 1. As shown in Fig 5A, the expressions of insulin receptor in STZ-treated group showed no significant difference (P>0.05), compared to control group. However, the mRNA expression of PI3K was significantly increased in the STZ-treated group compared to the control (Fig 5B). Furthermore, the expression of AKT and phosphorylation of AKT were detected by Western Blot. As shown in Fig 5C and 5D, there was a significant increase of the phosphorylation of AKT in STZ-treated groups (P < 0.01), and a significant increase of PI3K in STZ-treated groups, which suggested that STZ-induced gestational diabetes promoted the expression of PI3K and its downstream activation of AKT. Taken together, these results indicated that STZ-induced gestational diabetes promotes the newborn offspring germ cell cyst breakdown and primordial follicle formation via upregulating the PI3K/AKT signaling pathway.

### Insulin rescues the abnormal development caused by STZ-induced gestational diabetes in newborn mice

**Fig 6:**
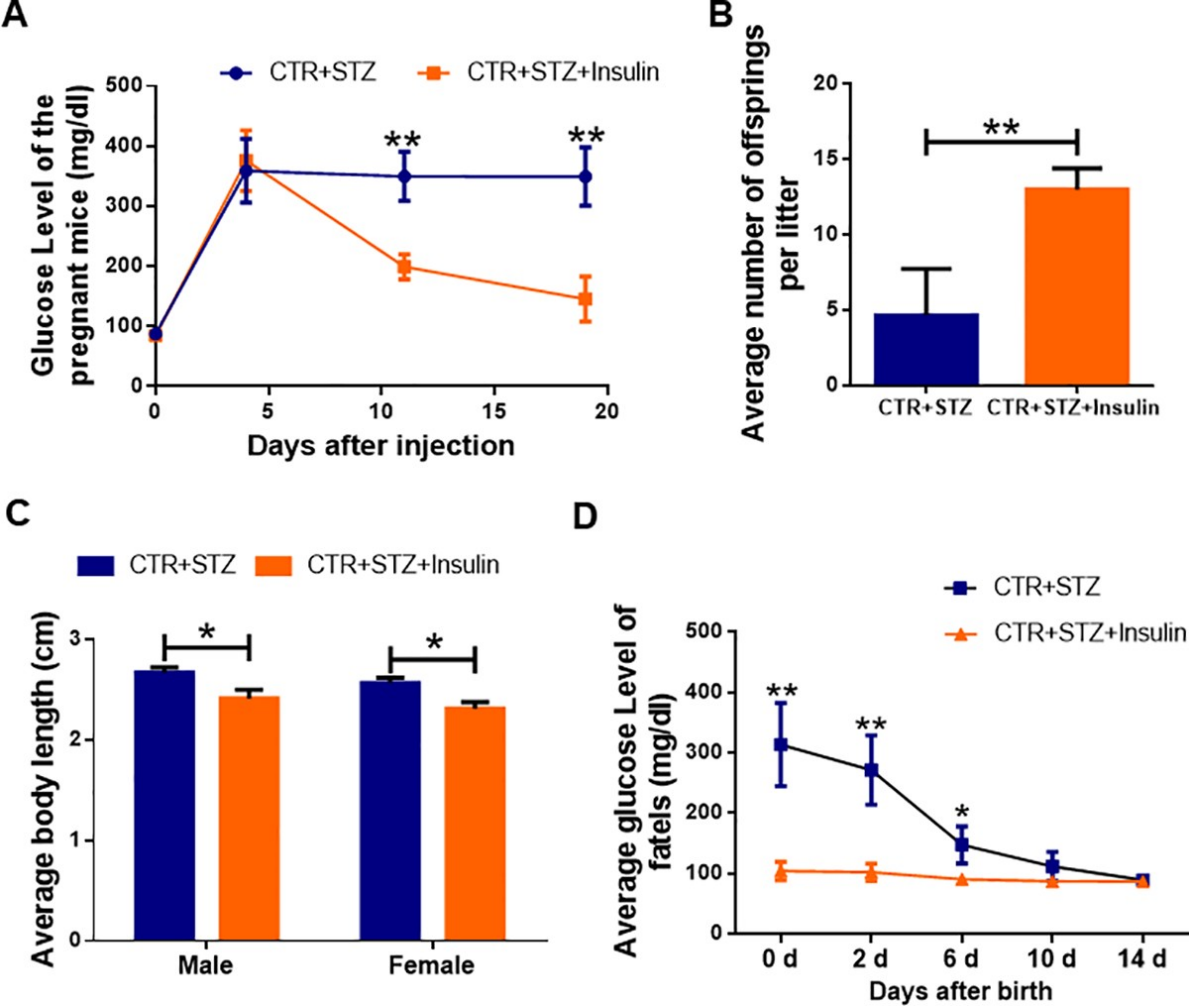
The effect of insulin treatment on the development of newborn mice. **(A)** The blood glucose levels in STZ-treated and STZ-Insulin-treated mice in 3 weeks. **(B, C)** The average number and length of newborn mice in control, STZ-treated and STZ-Insulin-treated groups. **(D)** The blood glucose levels of newborn mice in STZ-treated and STZ-Insulin-treated groups. At least 6–8 mice were included in each group.

Insulin is one of the most commonly used treatments for T1D. In order to properly carry out a complete pregnancy, diabetic women use the insulin treatment and other means to control their blood sugar level during pregnancy. We found that glucose levels of pregnant mice were significantly lower in the STZ-Insulin-treated group comparing to STZ-treated group, which showed that the increasing glucose concentrations in maternal diabetic mice are rescued by insulin treatment (P < 0.01) (Fig 6A). Therefore, we used insulin to treat diabetic pregnant mice in our study to examine whether the abnormal development observed in newborn mice could be rescued. The average number of offspring was 13±1.4 in STZ-Insulin-treated group, which is significantly higher than that (4.2±3.2) in STZ-treated group (P < 0.01) (Fig 6B). As shown in Fig 6C, our statistics analysis found that the average length of newborn mice was significantly shorter in STZ-Insulin-treated group comparing to STZ-treated group (P < 0.05). We also found that fetal glucose levels were significantly lower in the STZ-Insulin-treated group comparing to STZ-treated group (P < 0.01) (Fig 6D). Our studies showed that insulin treatment significantly increased the number of newborn mice, and lowered blood glucose levels, indicating that insulin can rescue the abnormal development in newborn mice caused by STZ-induced gestational diabetes.

### Insulin restores follicle development in newborn mice caused by STZ-induced gestational diabetes

**Fig 7:**
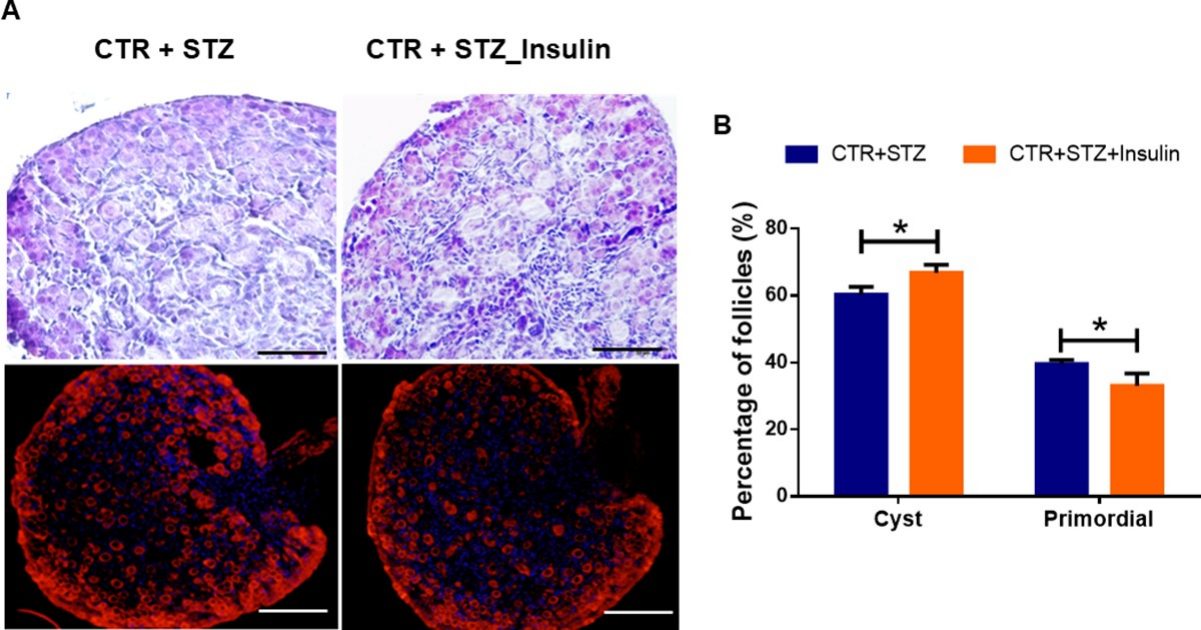
Insulin restores abnormal follicle development in newborn mice caused by STZ-induced gestational diabetes. **(A)** The histological appearance (Scale bars: 50 μm) and ovary whole-mount substratesimmunofluorescence of the newborn mouse ovarian tissue(Scale bars: 100 μm) in control and STZ-Insulin-treated groups. **(B)** It showed the percentage of oocytes in cyst and primordial follicle in STZ-treated and STZ-Insulin-treated groups at PND 1. At least 5 mice were analyzed in each group; Date are presented as mean ± SEM. *shows a significant difference between STZ-treated and control at P < 0.05; ** shows a significant difference at P < 0.01.

We showed that STZ-induced gestational diabetes promotes germ cell cyst breakdown and primordial follicle formation in newborn mice by upregulating the PI3K/AKT signaling pathway. Therefore, we used insulin to treat diabetic pregnant mice in our study to examine whether it can restore follicle development in newborn mice. HE staining and ovaryimmunofluorescence showed that no obvious change in total oocytes was observed in STZ-Insulin-treated and STZ-treated groups. However, there was a significant difference on oocytes in the cyst or primordial follicles. The percentages of unassembled follicles and primordial follicles were 60.39 ± 2.33% and 39.61 ± 1.25%, respectively, in STZ-treated group; while they were 66.90 ± 2.41% and 33.10 ± 3.65%, respectively, in STZ-Insulin-treated group (Fig 7A and 7B). The percentage of primordial follicles was significantly higher in STZ-treated group comparing to STZ-Insulin-treated group (P < 0.05). HE staining and ovaryimmunofluorescence showed that insulin treatment inhibited germ cell cyst breakdown and primordial follicle formation caused by STZ treatment.

**Fig 8:**
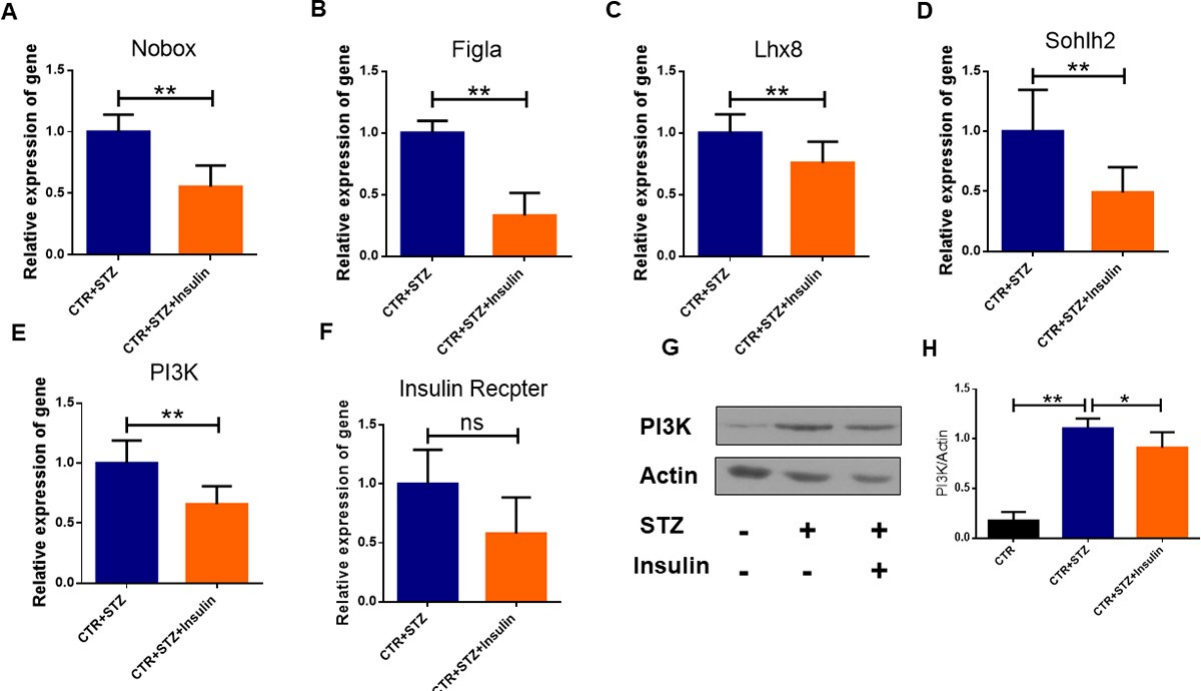
Insulin restores abnormal follicle development by regulates the PI3K/AKT signaling pathway in newborn mouse ovaries. **(A-F)** Real-time PCR analysis of mRNA expression levels of Nobox, Figla, Sohlh2, PI3K and Insulin and Receptor in STZ-treated and Insulin-STZ-treated groups. **(G-H)** The expression of PI3K protein was analyzed by western blot. Expression of β-actin was used as a loading control. At least 5 mice were analyzed in each group; Date are presented as mean ± SEM. * shows a significant difference between STZ-treated and control at P < 0.05; ** shows a significant difference at P < 0.01.

The relative mRNA expression levels of oocyte-specific genes including Nobox, Figla, Lhx8, Sohlh2 and PI3K in newborn mouse ovaries were significantly lowered by insulin treatment (P<0.01) (Fig 8A–8E). As shown in Fig 8F, the expressions of insulin receptor in Insulin-STZ-treated group showed no significant difference (P>0.05), compared to the STZ-treated group. As shown in Fig 5C and 5D, comparing to STZ-treated group, there was a significant decrease of AKT activation (P < 0.01), and a significant decrease of PI3K in in STZ-Insulin-treated groups (Fig 8G and 8H). These results showed that insulin treatment completely restores the PI3K activation caused by STZ-induced diabetes.

## Discussions

In this study, we provided new insights into the effect of gestational diabetes on the early stages of mammalian folliculogenesis. Previous studies have demonstrated that maternal diabetes adversely affects pregnancy outcomes, such as infertility, miscarriage, offspring with congenital malformations and fetal death [6]. Our results which are consistent with the previous studies in addition demonstrate that gestational diabetes adversely affects the development of the newborn offspring. The average number of offspring was reduced, and the average body length was significantly larger in the STZ-treated group compared to the control. Moreover, we found that offspring glucose and serum insulin level were significantly higher, but returned to normal levels in two weeks. The glucose from pregnant diabetic mice can be transferred into the offspring, resulting in the increase of offspring glucose level. The high glucose level in offspring may stimulate the growth of the pancreas, which in turn generates more insulin to decrease glucose level.

In our study, we analyzed the effect of STZ-induced gestational diabetes on follicle development in newborns. We injected the pregnant mice with STZ on day 2 of pregnancy and founded that STZ-induced diabetes promotes germ cell cyst breakdown and primordial follicle formation in newborns. No obvious change in total oocytes was observed between STZ-treated and control groups. Our results showed that the percentage of primordial follicles is around 39.87% in diabetic group, which is significantly higher than the control group. Oocyte-specific genes such as Nobox, Figla, Lhx8, and Kitl which play important roles in early follicular development are necessary to induce primordial follicle development and initiate folliculogenesis [28–30]. The mRNA expression of these genes was increased in STZ-treated group, demonstrating that the high mRNA expression of these oocyte-specific genes promoted offspring primordial follicle assembly in newborns.

Previous studies have shown that maternal diabetes adversely affects oocyte maturation and embryo development [6, 15]. However, our study provides additional insights into the effects of maternal diabetes on the fetal follicle development. Consistent with previous studies showing that the PI3K/AKT signaling pathway is crucial for primordial follicular assembly and activation process [31], we found that the PI3K expression in diabetic offspring mice is significantly higher than that in control mice. In diabetic offspring mice, AKT phosphorylation is also at a higher level. The high serum insulin level in offspring combining with the higher expression level of insulin receptor induces higher expression of PI3K and AKT activation in ovaries of diabetic newborns. These data suggested that the upregulated PI3K/AKT signaling pathway leads to primordial follicle assembly in diabetic mice. Since follicle assembly also requires additional signaling pathways, such as genes for growth factors and their receptors [32] and oocyte-specific transcription factors [33], further studies are required to examine their involvement. Our results also showed that insulin treatment could partially reverse the deleterious effects of diabetes on offspring follicle development.

## Conclusions

Our data supported that the PI3K/AKT signaling pathway regulates the early stage of folliculogenesis, resulting in promoting cyst breakdown and primordial follicle formation in newborn mice caused by STZ-induced gestational diabetes. These delirious effects can be rescued by insulin supplement. Our study suggested therapeutic potential of insulin to treat developmental defects in newborns suffering from gestational diabetes.
